# Exploring digital health literacy clusters in a Norwegian stroke survivor population—A cross-sectional study (NORFAST)

**DOI:** 10.1177/20552076251380049

**Published:** 2025-09-30

**Authors:** Anne-M. Linnestad, Ingrid Johansen Skogestad, Caryl L. Gay, Christine Råheim Borge, Bent Indredavik, Jan Stubberud, Anners Lerdal

**Affiliations:** 1 Department of Public Health and Interdisciplinary Health Sciences, Institute of Health and Society, Faculty of Medicine, 6305 University of Oslo, Oslo, Norway; 2 Department of Research, 60503 Lovisenberg Diaconal Hospital, Oslo, Norway; 3 Faculty of Health Science, Institute of Nursing, 87368 VID Specialized University, Oslo, Norway; 4 School of Nursing, 8785 University of California San Francisco, San Francisco, CA, USA; 5 Department of Neuromedicine and Movement Science, Faculty of Medicine and Health Science, 8018 Norwegian University of Science and Technology, Trondheim, Norway; 6 Department of Medical Quality Registries, St Olavs Hospital, Trondheim University Hospital, Trondheim, Norway; 7 Department of Psychology, 6305 University of Oslo, Oslo, Norway

**Keywords:** Digital health literacy, eHealth literacy, stroke, rehabilitation, cross-sectional, cluster analysis

## Abstract

**Background:**

Digital health technologies play a pivotal role in stroke prevention and rehabilitation. However, variations in digital health literacy (DHL) among stroke survivors may impede effective use of these tools. Understanding DHL-profiles and their influencing factors is essential for tailoring interventions.

**Aim:**

This study aimed to identify DHL-profiles among stroke survivors and possible associations with sociodemographic and clinical characteristics.

**Methods:**

We conducted a cross-sectional study with 177 first-time stroke patients (2021–2022) from the Norwegian stroke registry. DHL was measured using the eHealth Literacy Questionnaire. Cluster analysis identified DHL profiles, and multinomial regression explored associations with sociodemographic and clinical variables.

**Results:**

Three distinct DHL-profiles emerged: Novices (26%), Cautious users (57%), and Navigators (17%). Novices exhibited notable DHL limitations, cautious users demonstrated moderate proficiency, and navigators displayed a high level of proficiency. Participants aged ≥67 years were five times more likely to be Novices compared to Cautious users. Lower education and cognitive functioning increased novice likelihood, whereas living alone decreased the chance of being a Navigator.

**Conclusions:**

Diverse DHL-profiles among stroke survivors highlight the need for personalized digital health services. Tailored interventions, developed through co-design and addressing specific DHL challenges, may improve equitable access and enhance usability, ultimately promoting health equity.

## Introduction

The rapid digitalization of healthcare has introduced a variety of tools, from telemedicine to mobile health apps, that provide greater access to health information and enable more personalized care. The World Health Organization's 2020–2025 strategy encourages the widespread adoption of these digital health solutions to enhance global wellbeing.^1^ However, to fully benefit from these advances, individuals need digital health literacy (DHL), a critical set of skills necessary for effectively navigating, understanding, and using digital health resources.^2^ This competency is particularly important in managing complex health needs, such as secondary stroke prevention, where health literacy directly impacts care outcomes.^1^

DHL is a multidimensional concept,^3,4^ encompassing the knowledge, motivation, and skills required to seek, understand, and effectively use digital health information and digital tools.^1,5–8^ These skills support everyday tasks such as booking appointments, tracking health, managing records, and communicating with health-care providers.^2,9^ DHL also demands critical thinking, assessing the credibility of online health information and safeguarding personal data.^7,10^

Disparities in DHL limit the benefits that digital tools can provide for self-management and access to care.^11–13^ Although such technologies have the potential to expand healthcare accessibility, little research has explored their effectiveness for patients facing DHL challenges.^14^

Individuals with chronic illnesses such as stroke who also have limited DHL tend to show poorer self-management and a weaker understanding of their health compared to peers with more advanced DHL.^9^ Thus, promoting DHL in these groups may support better self-management and health behaviors.^11,12^ A simple “high-versus-low” label, however, masks substantial heterogeneity in DHL,^13,14^ and may conceal wide variations in motivation, skills, access, and cognitive capacity. Therefore, identifying nuanced DHL profiles, especially within specific chronic conditions like stroke, is essential for designing effective, equitable digital health interventions.^15^ For example, a patient may be highly motivated and digitally skilled but lack access to digital devices or the internet.

Stroke survivors face additional challenges due to post-stroke cognitive, language, and motor impairments, which further complicate the use of digital health tools.^16^ Nevertheless, this population stands to benefit significantly from digital innovations: telemedicine and online rehabilitation reduce physical barriers, support adherence, and foster self-efficacy.^16^ Yet disparities in DHL by age, socioeconomic status, and education exacerbate inequalities, with older adults and resource-limited groups most at risk of exclusion.^17,18^

Despite its importance, DHL in the context of stroke survivors has not been thoroughly examined. Latent-profile analyses to date have been conducted only in Chinese cohorts.^14^ Thus, there remains a notable lack of investigation into these profiles within Western society settings, leaving an important gap in understanding their unique characteristics and needs.

Addressing this gap, the present study aims to identify distinct DHL-profiles among Norwegian stroke survivors and explore potential associations with key sociodemographic and clinical characteristics.

## Methods

### Study design

This cross-sectional study was undertaken following the principles of an observational study.^19^

### Study population

Patients admitted to a Norwegian hospital in 2021–2022 for stroke care were recruited between June 2023 and May 2024 from a list of 2000 patients registered consecutively from the Norwegian Stroke Registry (NHR).^20^ NHR is a national medical quality registry which has a coverage rate of approximately 90% of all stroke cases admitted to hospitals in Norway.^20^

A sample of patients was prescreened and selected from the NHR based on the following eligibility criteria: first-ever stroke, pre-stroke modified Rankin Scale (mRS) score of 0 to 2, age 18 to 80 years in 2021–2022 (year the NHR sample was selected) and a valid mail address. The upper age limit was applied to minimize the effects of age-related cognitive, sensory, and physical impairments on the study results, and due to well-documented limitations in DHL among adults over 80 years.^21^ The following additional eligibility criteria were applied to the NHR sample prior to or during subsequent phone screening, sufficient cognitive ability to provide consent and ability to communicate by phone.

**Figure 1.**
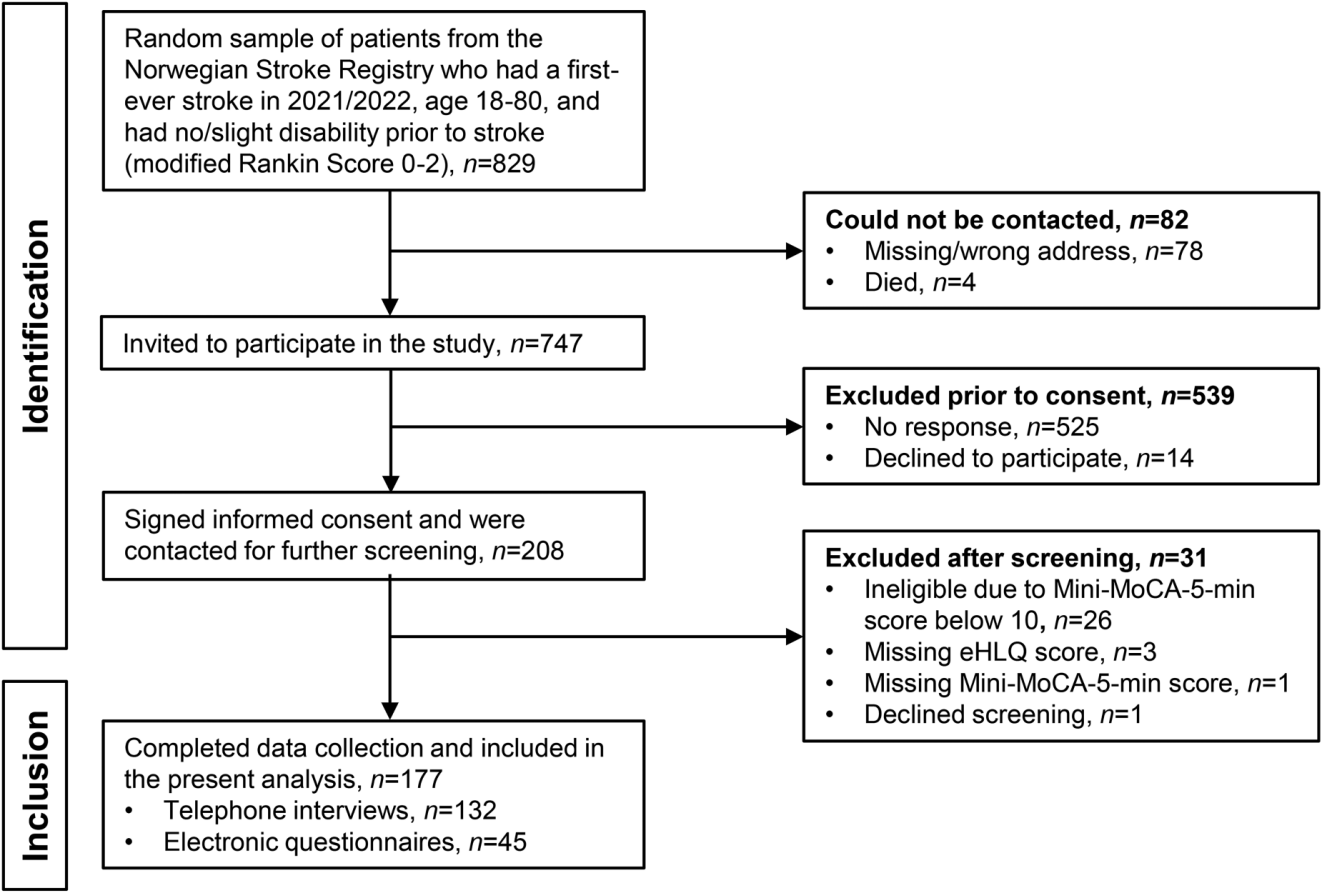
Flow chart of patient inclusion.

From the NHR sample, 829 patients were randomly selected to be contacted about the study (see Figure 1). Information about the study and a consent form were mailed by author A—ML to all living patients with a valid mail address. Patients were asked to return the consent form in a prepaid envelope and include a telephone number for further eligibility screening.

### Data collection and procedures

Data were collected from the NHR, telephone screening, and questionnaires, which were completed either electronically or in structured telephone interviews, depending on the patient's preference. All patients who returned their signed consent form were contacted by phone to complete the screening of cognitive function. Eligible patients were then asked to continue with the rest of the interview. For the questionnaire, patients were given the choice of completing it in a telephone interview or electronic survey. Data collection procedures were pilot tested with ten patients to familiarize the team with the questions and minimize potential data collection errors.

### Measures

#### Primary outcome

We assessed DHL with the eHealth Literacy Questionnaire (eHLQ).^3^ The eHLQ contains seven domains representing different dimensions of DHL: 1—*Using technology to process health information*, 2—*Understanding of health concepts and language,* 3*—Ability to actively engage with digital services,* 4—*Feel safe and in control,* 5—*Motivated to engage with digital services,* 6—*Access to digital services that work*, and 7—*Digital services that suit individual needs*. Each eHLQ dimension includes four-to-six items, with four response options: strongly disagree, disagree, agree, and strongly agree.^3^ The presence of the seven-factor structure and supportive validity evidence has been confirmed in previous studies.^3,4,22–25^

#### Other measures

The Norwegian version of the Mini Montreal Cognitive Assessment (Mini-MoCA, 5 min) version 2.1^26,27^ was used to objectively screen for cognitive impairment. The Mini-MoCA evaluates attention, verbal learning, memory, executive function and orientation, and can be administered reliably by telephone.^26,28^ Scores range from 0 to 15, with a score ≤11 typically indicating cognitive impairment.^28^ Although the English version of the Mini-MoCA has been validated for stroke patients and recommended for cognitive screening,^26,28–31^ current data supporting a cutoff of ≤11 have not been fully published.^28^ Because that threshold has not yet been formally validated in a Norwegian stroke cohort and our 10-patient pilot indicated several false-positive exclusions, we adopted a slightly lower cut-off of ≤10 to reduce the risk of mistakenly excluding individuals with adequate cognition. Patients scoring ≤10 were considered to have insufficient cognitive ability to provide informed consent and were subsequently excluded from the study. Patients were required to be in a quiet, distraction-free environment and have a reliable telephone connection to engage in testing.^32^

We included the following data from the NHR: neurological status and stroke severity (national institutes of health stroke scale, NIHSS),^33,34^ functional status (mRS),^35^ both pre-stroke and on day 7 post-stroke, independence in activities of daily living (Barthel Index),^36^ aphasia and stroke type at admission.

All sociodemographic variables were patient-reported and included age, sex, education, marital status, cohabitation status, and work status.

### Statistical analysis

All analyses were performed using SPSS, version 29.0.0.0 (IBM Corp, Armonk, NY)^37^ and Stata SE, version 17.0 (StataCorp, College Station, TX). Two-tailed p-values < 0.05 were considered statistically significant.

We employed a hierarchical explorative cluster analysis strategy to identify cluster solutions with Ward’s method for linkage,^38^ based on distance connectivity.^39^ This analysis identifies distinct groups of patients that share similar DHL profiles and may reveal variations that are not evident in average scores. Following standard guidelines,^37^ we selected the most parsimonious solution (i.e. the one with the fewest clusters) that demonstrated sufficient cluster homogeneity, as indicated by standard deviations <0.6 for each domain’s mean score.^39^ We also examined and compared the patterns generated by each consecutive cluster split. We identified differences between each new pattern and its parent cluster until further splits no longer revealed new or meaningful patterns in the data. Patients with missing DHL-values were excluded from the analysis.

To compare sociodemographic and clinical characteristics across clusters, we used Chi-square tests for categorical variables and one-way analysis of variance (ANOVA) for continuous variables. When Chi-square tests were significant, we used pairwise Z-tests to determine which clusters differed. When the ANOVA results were significant, we performed Scheffé post hoc tests for pairwise comparisons. We used the nonparametric Mann-Whitney U test or Kruskal-Wallis test for measures with non-normal distributions.

Multinomial logistic regression analysis was used to identify patient characteristics associated with DHL profiles in both unadjusted and adjusted models. Variables with p*-*values <0.1 in the unadjusted analyses were included in the adjusted model, controlling for both sex and age. In the case of collinearity (Pearson r ≥ 0.60) between potential covariates, the one with the stronger unadjusted association was included. The strength of associations was expressed as relative risk ratios with 95% confidence intervals.

## Results

Of the 829 patients in the NHR random sample who met the initial eligibility criteria, 747 (90%) were contactable and invited to participate. Of these, 208 (28%) consented to participate and were subsequently contacted for cognitive screening, and if found eligible, for data collection.

Of those contacted, 31 were excluded due to cognitive ineligibility, missing eHLQ data, or declining screening, resulting in a final sample of 177 participants (Figure 1). The characteristics of the study participants are shown in Table 1.

**Table 1.** Participant demographic and clinical characteristics.

Characteristics	N*	*n* (%)	Mean (*SD*)	Median (range)
***Demographics***				
Age, years	177		69.0 (9.8)	71 (35–84)
≥ 67		119 (67%)		
Sex, female	177	56 (32%)		
Education	176			
High school or less		84 (48%)		
College or university		92 (52%)		
Cohabitation status, living alone	177	52 (29%)		
Work status, paid work	176	54 (31%)		
				
***Clinical characteristics***				
Stroke type**	177			
Cerebral hemorrhage (i.61)		10 (6%)		
Cerebral infarct (i.63)		166 (94%)		
Unspecified (i.64)		1 (<1%)		
Stroke severity**				
NIHSS at hospital admission	159		2.95 (3.85)	1.0 (0–22)
0 = No stroke symptoms		49 (28%)		
1–4 = Mild stroke		71 (40%)		
5–15 = Moderate stroke		37 (21%)		
16–20 = Moderate to severe stroke		1 (<1%)		
21–42 = Severe stroke		1 (<1%)		
Missing		18 (10%)		
NIHSS at hospital discharge	147		0.81 (2.04)	0 (0–17)
No stroke symptoms (0)		101 (57%)		
1–4 = Mild stroke		38 (21%)		
5–15 = Moderate stroke		7 (4%)		
16–20 = Moderate to severe stroke		1 (<1%)		
21–42 = Severe stroke		0 (0%)		
Missing		30 (17%)		
Disability**				
mRS prior to stroke (scores >2 were ineligible)	177		0.26 (0.55)	0.00 (0–2)
mRS post-stroke day 7	177		1.12 (1.06)	1.00 (0–4)
Aphasia, present**	177	29 (16%)		
Activities of daily living**				
Barthel index day 7	105		90.9 (21.6)	100 (10–100)
Cognitive function	177		12.1 (1.5)	12.0 (10–15)
Mini-MoCA10-11 (Low)		68 (38%)		
Mini-Moca12-15 (High)		109 (62%)		

* Lower sample sizes for some variables due to missing data from the stroke registry or unanswered questions in the questionnaire.

**Clinical data from the National Stroke Registry.

mRS = Modified Rankin Scale (0–6, higher = more disability); NIHSS = National Institutes of Health Stroke Scale (0–42, higher = worse); Aphasia; Barthel Index (0–100, higher = better); Mini-MoCA = Mini Montreal Cognitive Assessment (0–15, higher = better).

### DHL in stroke survivors and naming of profiles

**Figure 2.**
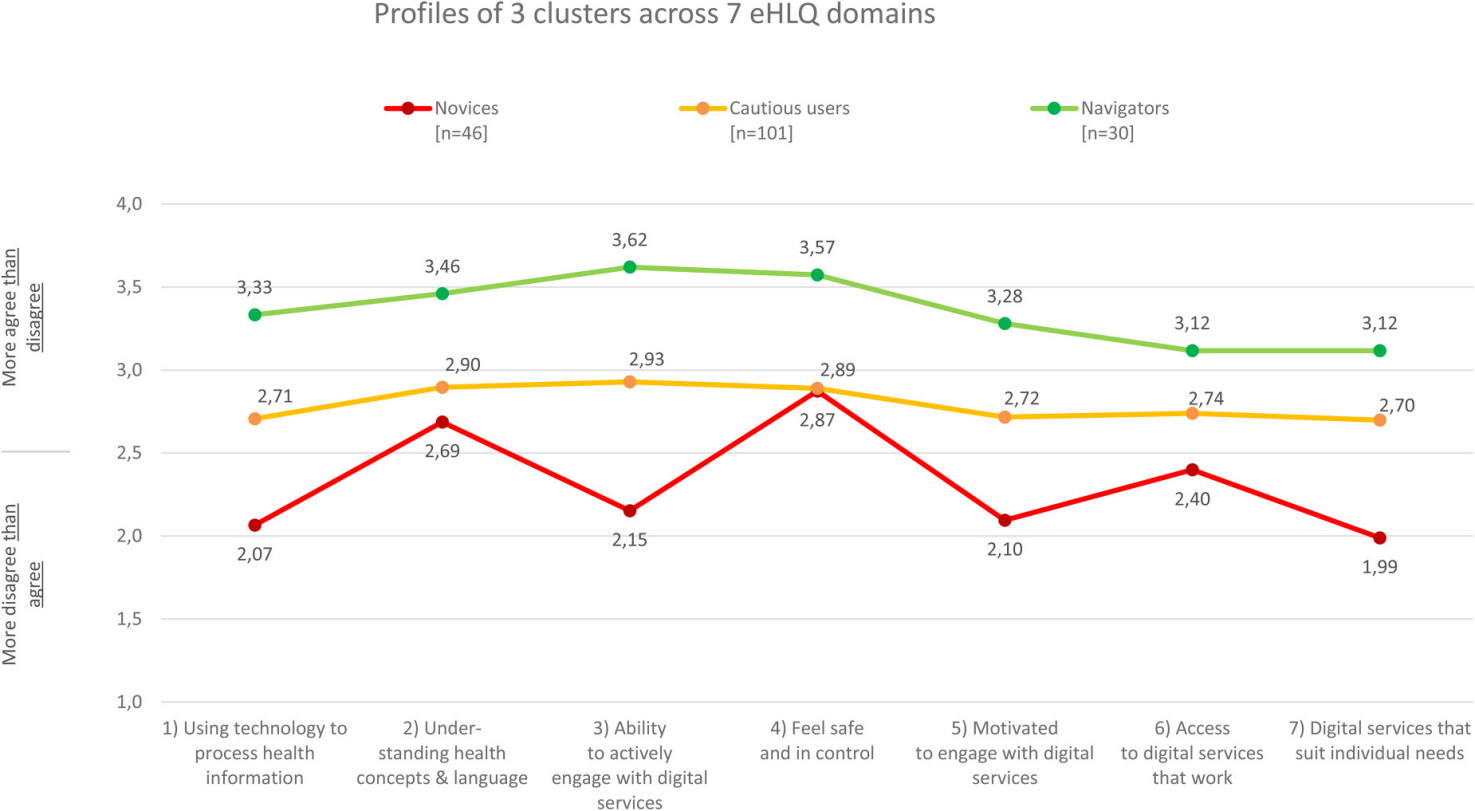
Three distinct DHL profiles of stroke survivors. The figure illustrates mean scores on each eHLQ domain for the three identified clusters, highlighting the differences in mean digital health literacy (eHLQ) scores between them. DHL: digital health literacy; eHLQ: eHealth Literacy Questionnaire.

Based on consideration of the statistical model and classification results of practical significance, interpretability and conciseness, the 3-cluster model was determined to be sufficient to describe the variation in DHL in this sample (see Figure 2). The cluster with the lowest and most variable domain scores was labeled “Digital Health Novices” (Novices) (*n* = 46), while the two clusters with higher and more consistent domain scores were labeled the “Cautious Digital Health Users” (Cautious users) (*n* = 101) and “Digital Health Navigators” (Navigators) (*n* = 30). All clusters differed significantly from each other on all eHLQ domains except that Novices and Cautious users did not differ on eHLQ Domain 4 (see Supplemental Table S1 for more details).

Solutions yielding more than three clusters were also examined. These generally resulted in one of the initial three clusters being split into two separate ones, with the other clusters remaining intact. Although these subsequent solutions identified more clusters, the overall patterns remained the same and did not reveal additional variation in DHL that was clinically meaningful.

Overall, the participants scored high on domain 4, *Feel safe and in control* and low on *Digital services that suit individual needs* (Figure 2; Table 2). Table 2 summarizes the characteristics of participants in each of the three clusters. Participants in the unique clusters differed with respect to participants’ age, education, cohabitation, work status, and cognitive function scores.

**Table 2.** Comparison of demographic and clinical characteristics across the three clusters (*n* = 177 unless indicated otherwise).

Characteristics	Digital health Novices (*n* = 46, 26%)	Cautious digital health users (*n* = 101, 57%)	Digital health Navigators (*n* = 30, 17%)	p-Value	Post hoc test
*Demographics*					
Age in years, mean (SD)	73.6 (7.6)	68.2 (9.7)	64.5 (10.7)	**<0**.**001**	1 > 2, 3
Age ≥ 67 years, % (*n*)	89% (41)	62% (63)	50% (15)	**<0**.**001**	1 > 2, 3
Female sex, % (*n*)	39% (18)	30% (30)	27% (8)	0.43	
College or university education, % (*n*)	33% (15)	56% (56)	70% (21)	**0**.**003**	1 > 2, 3
Living alone, % (*n*)	39% (18)	31% (31)	10% (3)	**0**.**022**	1 > 3
Not in paid work, % (*n*)	91% (42)	66% (66)	47% (14)	**<0**.**001**	1 > 2, 3
					
*Clinical characteristics*					
Stroke symptoms at discharge (NIHSS score), mean (SD) [*n* = 147]	0.89 (1.64)	0.94 (2.41)	0.24 (0.52)	0.31	
Stroke symptoms at discharge (NIHSS >0), % (*n*) [*n* = 147]	34% (12)	33% (29)	20% (5)	0.41	
Disability on post-stroke day 7 (mRS score), mean (SD)	1.15 (1.05)	1.14 (1.12)	1.03 (0.89)	0.88	
Disability on post-stroke day 7 (mRS ≥3), % (*n*)	11% (5)	11% (11)	3% (1)	0.49*	
Aphasia, % (*n*) [*n* = 174]	9% (4)	19% (19)	20% (6)	0.27	
ADL (Barthel Index-day 7), mean (SD) [*n* = 105]	91.1 (20.6)	90.5 (21.8)	92.1 (23.7)	0.96	
Cognitive function (Mini-MoCA score), mean (SD)	11.4 (1.4)	12.3 (1.4)	12.4 (1.5)	**0**.**002**	1 < 2, 3
Low normal cognitive function (Mini-MoCA < 12), % (*n*)	54% (25)	34% (34)	30% (9)	**0**.**033**	ns

* Fisher's exact test (all other comparisons of categorical variables use Chi-square tests and comparisons of continuous variables use one-way ANOVA with Scheffé post hoc tests).

ANOVA: analysis of variance; ns: no significant post hoc tests; MoCA: Montreal Cognitive Assessment; mRS: modified Rankin Scale; NIHSS: National Institutes of Health Stroke Scale.

### Associations between digital health literacy profiles and participant characteristics

The regression analyses, using Cautious users as the reference group, showed that participants who are older, have a lower level of education, or have a lower level of cognitive function are more likely to be a Novice and less likely to be a Cautious user, controlling for sex and cohabitation (Table 3). Older participants had a five-fold higher risk of being a Novice than a Cautious user. Those who live alone are significantly less likely to be a Navigator and more likely to be a Cautious user, controlling for sex, age, level of education and level of cognitive function.

**Table 3.** Multinomial regression analyses: unadjusted and adjusted associations between stroke survivor characteristics and three digital health literacy clusters.

Variables	Cluster 1: Novices vs Cautious users				Cluster 3: Navigators vs Cautious users		
	RR	95% CI	p-Value		RR	95% CI	p-Value
**Unadjusted models**							
*Demographic characteristics*							
Female sex	1.52	0.73–3.16	0.26		0.86	0.34–2.15	0.75
Age ≥ 67 years	**4**.**95**	**1.80–13.6**	**0**.**002**		0.60	0.27–1.37	0.23
College/university education	**0**.**38**	**0.18–0.79**	**0**.**010**		1.83	0.76–4.40	0.18
Living alone	1.45	0.70–3.01	0.32		**0**.**25**	**0.07–0.89**	**0**.**032**
In paid work	**0**.**18**	**0.06–0.56**	**0**.**003**		2.22	0.97–5.08	0.059
*Clinical characteristics*							
Stroke symptoms (NIHSS)*	0.99	0.82–1.19	0.90		0.60	0.31–1.15	0.12
Disability (mRS on day 7)	1.01	0.73- 1.40	0.94		0.91	0.61–1.35	0.63
Aphasia	0.41	0.13–1.29	0.13		1.05	0.38–2.93	0.92
ADL (Barthel Index)	1.00	0.98–1.02	0.90		1.00	0.98–1.03	0.77
Mini-MoCA score	**0**.**65**	**0.50–0.85**	**0**.**001**		1.07	0.81–1.42	0.63
Adjusted model**							
Female sex	1.89	0.82–4.35	0.13		0.97	0.37–2.52	0.94
Age ≥ 67 years	**5**.**56**	**1.87–16.5**	**0**.**002**		0.60	0.24–1.46	0.26
College/university education	**0**.**32**	**0.14–0.73**	**0**.**007**		2.17	0.86–5.51	0.10
Living alone	1.24	0.55- 2.79	0.61		**0**.**27**	**0.08–0.999**	**0**.**0499**
Mini-MoCA score	**0**.**70**	**0.52–0.95**	**0**.**020**		1.02	0.77–1.37	0.87

Reference group = Cluster 2 – Cautious users, the largest cluster.

* At discharge.

** Adjusted analyses included sex, age, and all covariates with p < .10 in unadjusted analyses; work was excluded due to collinearity with age

CI: confidence interval; RR: Relative Risk ratio; ADL: Activities of Daily Living; MoCA: Montreal Cognitive Assessment; mRS: modified Rankin Scale; NIHSS: National Institutes of Health Stroke Scale.

### Representativeness

The 177 study participants included in this analysis differed significantly from the 570 patients who were excluded. Compared to those who were excluded, the included patients were younger, were more likely to be male, were more likely to have an ischemic stroke and less likely to have a hemorrhagic stroke, had better functional status (mRS and Barthel Index) on day 7 following their stroke, and had better stroke severity scores (NIHSS) at discharge. However, the included and excluded patients had similar rates of aphasia, and did not differ with respect to the location of their stroke or stroke severity at admission (NIHSS). Group comparisons were performed using χ² tests for categorical variables, t-tests for normally distributed continuous variables (with adjustments for unequal variances where appropriate), and Mann–Whitney U tests for skewed distributions such as NIHSS scores; full results are presented in Supplemental Table S2.

## Discussion

This nationwide, population-based study is among the first to classify stroke survivors from a European national stroke registry into distinct DHL profiles, derived from a multidimensional assessment of DHL. Three groups were identified: Novices, Cautious users, and Navigators, each displaying unique sets of strengths and challenges. These profiles differed significantly by age, education, living situation, and cognitive function. Our findings are in line with prior research showing that older adults typically exhibit lower DHL^10,11,13,40^ and further highlight that stroke survivors with lower educational attainment are particularly at risk of reduced DHL.

The Novices profile was characterized by the lowest competence in processing health information digitally, likely reflecting age-related cognitive decline and limited search-and-appraisal skills.^40^ Low levels of confidence and motivation further hinder digital engagement, despite adequate access to services.^3,41^ The Novice profile highlights the obstacles faced by older adults when engaging with digital health technologies.^13,40,42,43^ However, Novices’ relative strengths in general health knowledge and trust in healthcare systems provide a foundation for targeted interventions. As this group also included individuals with initial aphasia and lower cognitive functioning, our findings suggest that tailored training, caregiver involvement, and stronger social support structures will be essential to mitigate barriers and enable the effective use of digital health services.^10,42,43^ Furthermore, the integration of accessibility features, such as simplified interfaces, multimodal content, and language support, may further enhance the usability and inclusiveness of such services for this vulnerable population.

Cautious users, the largest subgroup, demonstrated moderate engagement and a selective approach to digital health tools.^44^ Their pattern of scores suggests awareness of potential benefits but also hesitancy in fully integrating digital services into care routines. Concerns about privacy, security, and service personalization appear to influence their balanced yet restrained engagement.^44^ Given their relatively high educational attainment, it is likely that digital skills are not the primary limiting factor^24^; rather, individual concerns and cognitive sequelae, including aphasia, may play a role.^40,42,43^ Addressing privacy and tailoring services to personal health contexts may enhance participation in this group.^44^

The Navigators, though the least common cluster, exhibited strong DHL across domains, demonstrating advanced ability to use, evaluate, and apply digital tools for health management. The strong performance in ability to actively engage with digital services may reflect advanced skills and a broad set of competencies, including critical evaluation and operational skills. These individuals were more likely to be younger and higher educated, aligning with evidence that such characteristics are associated with stronger DHL.^10,14^ However, the presence of aphasia at onset and a moderate cognitive function highlights potential cognitive challenges even among the most adept users.^40,42,43^ Nevertheless, their comparatively lower scores in motivation and access to individualized services may indicate that even proficient users may benefit from more customizable digital solutions.^14^ This emphasizes the importance of designing flexible, relevant digital health services to maintain motivation and ensure continued engagement.^44^

The identification of DHL profiles in stroke survivors has direct clinical relevance. Given the high prevalence of cognitive and communication impairments, including aphasia, interventions must go beyond general digital literacy training. Simplified digital content, audio-visual formats, alternative communication strategies, and involvement of caregivers can expand accessibility for those with impairments.^41–43^ For Novices, resources should focus on building fundamental confidence and motivation, leveraging trust in healthcare professionals as a pathway into digital engagement. Cautious users may benefit from reassurance around privacy, security, and selective personalization, while Navigators require tools that remain engaging and aligned with individual health goals.

These tailored approaches may support self-management, enhance access to telehealth, and strengthen secondary prevention efforts, thereby improving long-term outcomes in this population. Integration of caregivers and support networks into digital health strategies is particularly important to ensure continuity and sustainability, especially for those with persistent cognitive or communicative limitations.

A major strength of this study is its population-based sample drawn from a national stroke registry, which yielded a nearly complete dataset across a diverse spectrum of survivors.^20^ Uniquely, individuals with aphasia and lower DHL levels were included by offering multiple response modes like mail, telephone, or electronic questionnaires, a methodological approach that reduces exclusion bias and enhances ecological validity.

Nevertheless, several limitations should be considered. The Mini-MoCA was administered via telephone, where background noise and lack of visual cues may have inflated error rates, particularly near the exclusion cutoff.^32^ The modest sample size (*n* = 177) may have limited statistical power and yielded small clusters, reducing stability and generalizability, especially for the Navigator group. However, these small clusters can reveal variations typically seen in clinical settings and can generate valuable hypotheses. Low response rate and sex differences raise the possibility of self-selection bias, whereby participants may differ in DHL from nonrespondents. Finally, the sample underrepresents individuals with more severe strokes, greater functional impairment, and higher age, meaning caution is required when extrapolating findings to these groups, who may also face the greatest barriers to digital engagement.

Future studies should replicate these findings in larger, more diverse cohorts to confirm cluster stability, better capture less represented populations, and test the impact of tailored interventions based on DHL profiles. Longitudinal observational work is warranted to examine how DHL evolves during recovery and cognitive changes after stroke. A critical next step is the design and evaluation of intervention strategies, including caregiver-supported, aphasia-friendly, and personalized digital health solutions, to determine their effectiveness in improving engagement, self-management, and secondary prevention outcomes.

## Conclusion

The rapid increase in online health information, along with the growing use of technology and telehealth services, requires a population skilled in evaluating information, avoiding misinformation, and accessing services effectively. By profiling digital health literacy in a national cohort of stroke survivors, we identified three distinct patterns, Novices, Cautious users, and Navigators. These profiles illustrate the range of DHL and its impact on health engagement and outcomes within a subgroup of patients, emphasizing the need for personalized digital health services and targeted interventions to improve DHL. The findings underscore the importance of user co-design in developing digital health solutions and highlight that enhancing DHL is crucial for promoting self-management and health equity among stroke survivors.

## Supplemental Material

sj-docx-1-dhj-10.1177_20552076251380049 - Supplemental material for Exploring digital health literacy clusters in a Norwegian stroke survivor population—A cross-sectional study (NORFAST) Supplemental material, sj-docx-1-dhj-10.1177_20552076251380049 for Exploring digital health literacy clusters in a Norwegian stroke survivor population—A cross-sectional study (NORFAST) by Anne-M. Linnestad, Ingrid Johansen Skogestad, Caryl L. Gay and Christine Råheim Borge, Bent Indredavik, Jan Stubberud, Anners Lerdal in DIGITAL HEALTH

sj-docx-2-dhj-10.1177_20552076251380049 - Supplemental material for Exploring digital health literacy clusters in a Norwegian stroke survivor population—A cross-sectional study (NORFAST) Supplemental material, sj-docx-2-dhj-10.1177_20552076251380049 for Exploring digital health literacy clusters in a Norwegian stroke survivor population—A cross-sectional study (NORFAST) by Anne-M. Linnestad, Ingrid Johansen Skogestad, Caryl L. Gay and Christine Råheim Borge, Bent Indredavik, Jan Stubberud, Anners Lerdal in DIGITAL HEALTH
