# Supplementary material for: Exploring digital health literacy clusters in a Norwegian stroke survivor population—A cross-sectional study (NORFAST)
Source: Digit Health. 2025 Sep 30;11:20552076251380049. doi: 10.1177/20552076251380049 (PMC12484892; doi:10.1177/20552076251380049)
Supplement: sj-docx-1-dhj-10.1177_20552076251380049 - Supplemental material for Exploring digital health literacy clusters in a Norwegian stroke survivor population—A cross-sectional study (NORFAST) [file sj-docx-1-dhj-10.1177_20552076251380049.docx]

Table S1. Comparison of digital health literacy (eHLQ) domain scores across the three clusters (N=177)

| **Digital health literacy domain from the eHLQ**  **(higher scores = higher ability)** | **Novices**  **(n=46, 26%)** | **Cautious Users**  **(n=101, 57%)** | **Navigators**  **(n=30, 17%)** | ***P*-value** | **Post-hoc**  **Test** |
| --- | --- | --- | --- | --- | --- |
| Domain 1:  Using technology to process health information | 2.07 (0.38) | 2.71 (0.30) | 3.33 (0.46) | <.001 | 1 < 2 < 3 |
| Domain 2:  Using of health concepts and language | 2.69 (0.38) | 2.90 (0.32) | 3.46 (0.35) | <.001 | 1 < 2 < 3 |
| Domain 3:  Ability to actively engage with digital services | 2.15 (0.47) | 2.93 (0.34) | 3.62 (0.38) | <.001 | 1 < 2 < 3 |
| Domain 4:  Feel safe and in control | 2.87 (0.28) | 2.89 (0.36) | 3.57 (0.46) | <.001 | 1, 2 < 3 |
| Domain 5:  Motivated to engage with digital services | 2.10 (0.39) | 2.72 (0.31) | 3.28 (0.37) | <.001 | 1 < 2 < 3 |
| Domain 6:  Access to digital services that works | 2.40 (0.32) | 2.74 (0.32) | 3.12 (0.50) | <.001 | 1 < 2 < 3 |
| Domain 7:  Digital services that suit individual needs | 1.99 (0.38) | 2.70 (0.30) | 3.12 (0.48) | <.001 | 1 < 2 < 3 |

Data in the table are means and standard deviations. *P*-values are from oneway analysis of variance (ANOVA). Scheffé post-hoc tests indicate that all clusters differ significantly from each other on all eHLQ domains except that clusters 1 and 2 do not differ on eHLQ Domain 4.
