# Supplementary material for: Exploring digital health literacy clusters in a Norwegian stroke survivor population—A cross-sectional study (NORFAST)
Source: Digit Health. 2025 Sep 30;11:20552076251380049. doi: 10.1177/20552076251380049 (PMC12484892; doi:10.1177/20552076251380049)
Supplement: sj-docx-2-dhj-10.1177_20552076251380049 - Supplemental material for Exploring digital health literacy clusters in a Norwegian stroke survivor population—A cross-sectional study (NORFAST) [file sj-docx-2-dhj-10.1177_20552076251380049.docx]

**Supplementary Table 2**. Comparison of patients included and excluded from the analysis (*n*=747 invited to participate ^a^)

| **Characteristics** | **Total sample** | **Excluded**  *n*=570 | **Included**  *n*=177 | **P-value** |
| --- | --- | --- | --- | --- |
| ***Demographics*** |  |  |  |  |
| Age in years, mean (SD) | *n*=747 | 70.9 (10.6) | 69.0 (9.8) | **.030** |
| Age group, n (%) | *n*=747 |  |  | .21 |
| ≥ 67 years | 530 (71.0%) | 411 (72.1%) | 119 (67.2%) |  |
| Sex, n (%) | *n*=747 |  |  | **.018** |
| Male | 454 (60.8%) | 333 (58.4%) | 121 (68.4%) |  |
| Female | 293 (39.2%) | 237 (41.6%) | 56 (31.6%) |  |
| ***Clinical characteristics*** |  |  |  |  |
| Stroke type, n (%) | *n*=745 ^b^ |  |  | **.031** |
| Cerebral hemorrhage | 74 (9.9%) | 64 (11.2%) | 10 (5.7%) |  |
| Cerebral infarct | 671 (90.1%) | 505 (88.8%) | 166 (94.3%) |  |
| Stroke location, n (%) | *n*=747 |  |  | .11 |
| Right | 267 (35.7%) | 210 (36.8%) | 57 (32.2%) |  |
| Left | 307 (41.1%) | 236 (41.4%) | 71 (40.1%) |  |
| Bilateral | 34 (4.6%) | 29 (5.1%) | 5 (2.8%) |  |
| Uncertain | 69 (9.2%) | 46 (8.1%) | 23 (13.0%) |  |
| Unknown | 70 (9.4%) | 49 (8.6%) | 21 (11.9%) |  |
| Stroke severity (NIHSS) |  |  |  |  |
| At hospital admission | *n*=664 |  |  |  |
| Median (IQR) | 2.0 (0.3, 5.0) | 2.0 (1.0, 5.0) | 1.0 (0.0, 4.0) | .058 ^c^ |
| Score category, n (%) |  |  |  | .052 |
| 0 = No stroke symptoms | 166 (25.0%) | 117 (23.2%) | 49 (30.8%) |  |
| 1 – 42 = Mild to severe stroke symptoms | 330 (49.7%) | 259 (51.3%) | 71 (44.7%) |  |
| At hospital discharge | *n*=607 |  |  |  |
| Median (IQR) | 0.0 (0.0, 2.0) | 0.0 (0.0, 2.0) | 0.0 (0.0, 1.0) | **<.001** ^c^ |
| Score category, n (%) |  |  |  | **<.001** |
| 0 = No stroke symptoms | 336 (55.4%) | 235 (51.1%) | 101 (68.7%) |  |
| 1 – 42 = Mild to severe stroke symptoms | 271 (44.6%) | 225 (48.9%) | 46 (31.6%) |  |
| Disability (mRS) | *n*=747 |  |  |  |
| Before stroke ^d^ |  |  |  |  |
| Mean (SD) | 0.36 (0.61) | 0.39 (0.62) | 0.26 (0.55) | **.008** ^e^ |
| Post-stroke day 7 |  |  |  |  |
| Mean (SD) | 1.51 (1.25) | 1.64 (1.27) | 1.12 (1.06) | **<.001** ^e^ |
| Disability category, n (%) |  |  |  | **<.001** |
| 0-2 = none to slight | 582 (77.9%) | 422 (74.0%) | 160 (90.4%) |  |
| 3-4 = moderate to moderately severe | 165 (22.1%) | 148 (26.0%) | 17 (9.6%) |  |
| Aphasia, n (%) | *n*=736 |  |  | .20 |
| No aphasia | 588 (79.9%) | 443 (78.8%) | 145 (83.3%) |  |
| Has aphasia | 148 (20.1%) | 119 (21.2%) | 29 (16.7%) |  |
| Activities of daily living (Barthel) | *n*=449 |  |  | **.001** ^e^ |
| Mean (SD) | 84.3 (27.0) | 82.3 (28.1) | 90.9 (21.6) |  |

NIHSS = National Institutes of Health Stroke Score (scale 0-42, higher = worse); mRS = Modified Rankin Scale (scale 0–6, higher= more disability); Barthel Index for Activities of Daily Living (scale 0-100, higher = more independent)

^a^ Excludes 82 patients who could not be invited to the study because the registry lacked a valid address, or they had died.

^b^ 2 patients with unspecified stroke type (1 included and 1 excluded) were omitted for this comparison due to their small number.

^c^ Due to the skewed distribution of scores, NIHSS scores are reported as medians with interquartile range (IQR) and analyzed using Mann-Whitney U tests.

^d^ Patients with pre-stroke mRS scores >2 were not eligible; therefore no included or excluded patients had pre-stroke mRS scores >2

^e^ Comparisons used separate variance t-test due to unequal variances
